# Supplementary material for: Voltage-dependent gating of SV channel TPC1 confers vacuole excitability
Source: Nat Commun. 2019 Jun 14;10:2659. doi: 10.1038/s41467-019-10599-x (PMC6572840; doi:10.1038/s41467-019-10599-x)
Supplement: Supplementary file 1 — Supplementary Information [file 41467_2019_10599_MOESM1_ESM.pdf]

SUPPLEMENTARY FIGURES

Supplementary Fig. 1

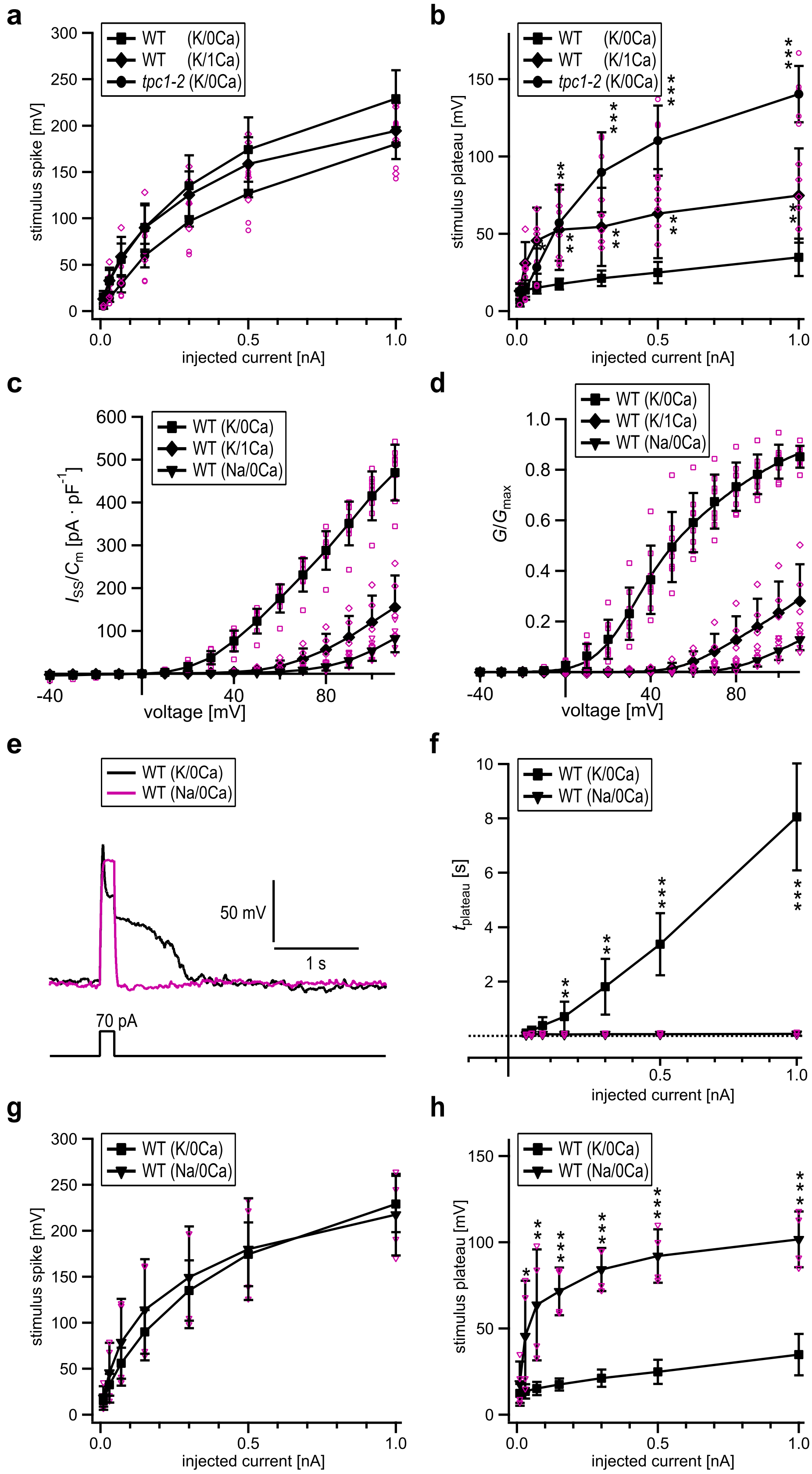

Supplementary Fig. 1: Effect of altered voltage-dependent SV/TPC1 channel gating on vacuolar electrical excitability. a, b, Amplitudes of stimulus spike (**a**) and stimulus plateau (**b**) plotted against the corresponding injected current. Current clamp experiments were carried out with mesophyll vacuoles from wild type (WT) and *tpc1-2* mutant plants under K^+-based solute conditions in the absence or presence of 1 mM luminal Ca^{2+} (K/0Ca and K/1Ca, respectively). Number of experiments in **a, b** was n = 7 for WT (K/0Ca, squares), n = 5 for WT (K/1Ca, diamonds) and n = 5 for *tpc1-2* (circles). In **b** significant differences between wild type (K/0Ca) versus wild type (K/1Ca) and wild type (K/0Ca) versus *tpc1-2* are displayed by stars according to the determined p-values (* = $p < 0.05$, ** = $p < 0.01$, *** = $p < 0.001$). **c, d,** Steady-state current density (I_{ss}/C_m, **c**) and normalized conductance (G/G_{max}, **d**) were plotted against the corresponding voltage. $G(V)$ data were described by Equation (1) (solid lines). Voltage clamp experiments in **c, d** were carried out with either standard K^+-based solutions in the absence or presence of 1 mM luminal Ca^{2+} (K/0Ca or K/1Ca, respectively) or Na^+-based bath/pipette solutions (Na/0Ca) without addition of Ca^{2+} to the pipette medium. The number of experiments was n = 8 for WT (K/0Ca, squares), n = 5 for WT (K/1Ca, diamonds) and n = 5 for WT (Na/0Ca, inverted triangles). The symbols in **d** refer to the same conditions as in **c**. Significant differences in the current density (**c**) and normalized conductance (**d**) between wild type (K/0Ca) versus wild type (K/1Ca) and wild type (K/0Ca) versus wild type (Na/0Ca) were determined at voltages ≥ +30 mV. **e,** Representative membrane voltage responses (upper panel) of wild type to 70-pA current injection (lower panel) under K^+- or Na^+-based solute conditions in the absence of luminal Ca^{2+}. The wild type voltage trace in **e** is identical to that shown in Fig. 1a. **f-h,** Lifetime of the post-stimulus plateau phase (**f**), amplitudes of stimulus spike (**g**) and stimulus plateau (**h**) derived for the experimental conditions from **e** were plotted against the corresponding injected current. The dotted line in **f** represents the zero line. The number of experiments was n = 7 for WT (K/0Ca, squares) and n = 4 for WT (Na/0Ca, inverted triangles). Wild type values (K/0Ca) in **a, b** and **f-h** are identical to those given in Fig. 1b, c. Closed symbols in **a-d** and **f-h** represent means ± standard deviation from experiments with individual vacuoles. In **a-d, f-h** individual data points are given as open magenta symbols for wild type (K/1Ca: diamonds, Na/0Ca: inverted triangles) and *tpc1-2* (circles). In **f** and **h** significant differences are displayed by stars according to the determined p-values (* = $p < 0.05$, ** = $p < 0.01$, *** = $p < 0.001$). Exact p-values determined for the data sets shown in **a-d** and **g** are listed in Supplementary Data 1 and 2. Statistical analysis in **a-d, f-h** was performed with one-way ANOVA followed by Bonferroni's post-hoc comparison test. Source data are provided as a Source Data file.

Supplementary Fig. 2

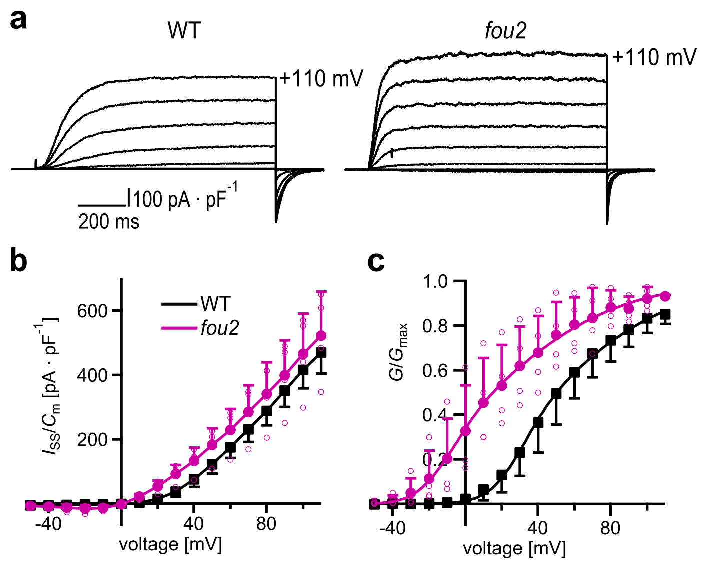

Supplementary Fig. 2: Voltage-dependent SV/TPC1 channel activity of wild type and *fou2* vacuoles. a, Current responses of wild type (WT) and *fou2* vacuoles to voltage pulses of 1 s duration, in the range of -70 to +110 mV applied in 20-mV steps from a holding voltage of -60 mV. **b, c,** Steady-state current density (I_{ss}/C_m, **b**) and normalized conductance (G/G_{max}, **c**) determined from current recordings as shown in **a** were plotted against the corresponding voltage. Data points of the $G/G_{max}(V)$ curves (**c**) were described by Equation (1) and fitted with the following parameters (solid lines): $V_{1\text{-WT}}$ = 4.3 mV, $V_{2\text{-WT}}$ = 13.8 mV, $V_{1\text{-}fou2}$ = -61.2 mV and $V_{2\text{-}fou2}$ = -10.7 mV. Voltage clamp experiments were carried out in K^+-based standard solutions without luminal Ca^{2+}. Closed black squares (WT) and closed magenta circles (*fou2*) in **b, c** represent means ± standard deviation from experiments with individual vacuoles. Individual data points (open magenta symbols) are given for *fou2*. The symbols in **c** refer to the same genotypes as in **b**. The number of experiments was n = 8 for wild type and n = 4 for *fou2*. Wild type values in **b, c** are identical to those given in Supplementary Fig. 1c, d (WT (K/0Ca)). Source data are provided as a Source Data file.

Supplementary Fig. 3

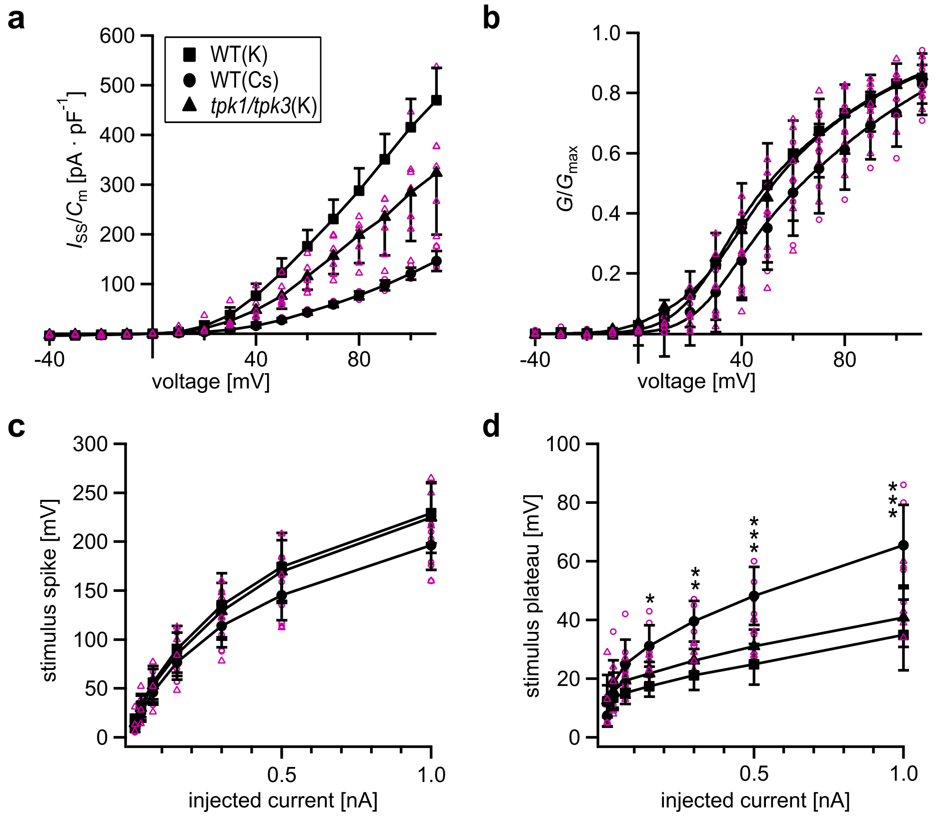

Supplementary Fig. 3: Effect of the K^+ channel blocker Cs^+ and the loss of TPK-type channel function on SV/TPC1 channel activity and membrane voltage response. a, b, Steady-state current density (I_{ss}/C_m, **a**) and normalized conductance (G/G_{max}, **b**) determined for wild type (WT) and *tpk1/tpk3* vacuoles were plotted against the corresponding voltage. Voltage clamp experiments were carried out either with K^+- or Cs^+-based solutions in the absence of luminal Ca^{2+} as referred to (K) or (Cs). Data points of all $G(V)$ curves (**b**) were described by Equation (1). Number of experiments was in **a, b** n = 8 for WT (K), n = 4 for WT (Cs) and n = 7 for *tpk1/tpk3* (K). In **a,** significant differences in the current density between wild type (K) versus wild type (Cs) were determined at voltages ≥ +40 mV. **c, d,** Amplitudes of stimulus spike (**c**) and stimulus plateau (**d**), derived for the same experimental conditions shown in **a, b** were plotted against the corresponding injected current. Number of experiments was in **c, d** n = 7 for WT (K), n = 7 for WT (Cs) and n = 6 for *tpk1/tpk3* (K). Closed symbols in **a-d** represent means ± standard deviation from experiments with individual vacuoles. Individual data points (open magenta symbols) are given for wild type (Cs, circles) and *tpk1/tpk3* (triangles). Wild type values (WT(K)) in **a, b** are identical to those given in Supplementary Fig. 1c, d (WT (K/0Ca)), and wild type values (WT(K)) in **c, d** are identical to those given in Fig. 1a, b (WT (K/0Ca)). Symbols in **a-d** have the following meaning: Closed squares refer to WT(K), closed/open circles to WT(Cs) and closed/open triangles to *tpk1/tpk3*(K). In **d,** significant differences between WT(K) and WT(Cs) are displayed by stars according to the determined p-values (* = p < 0.05, ** = p < 0.01, *** = p < 0.001; one-way ANOVA followed by Bonferroni's post-hoc comparison test). Exact p-values determined for all data sets (**a-d)** are listed in Supplementary Data 1 and 2. Source data are provided as a Source Data file.

Supplementary Fig. 4

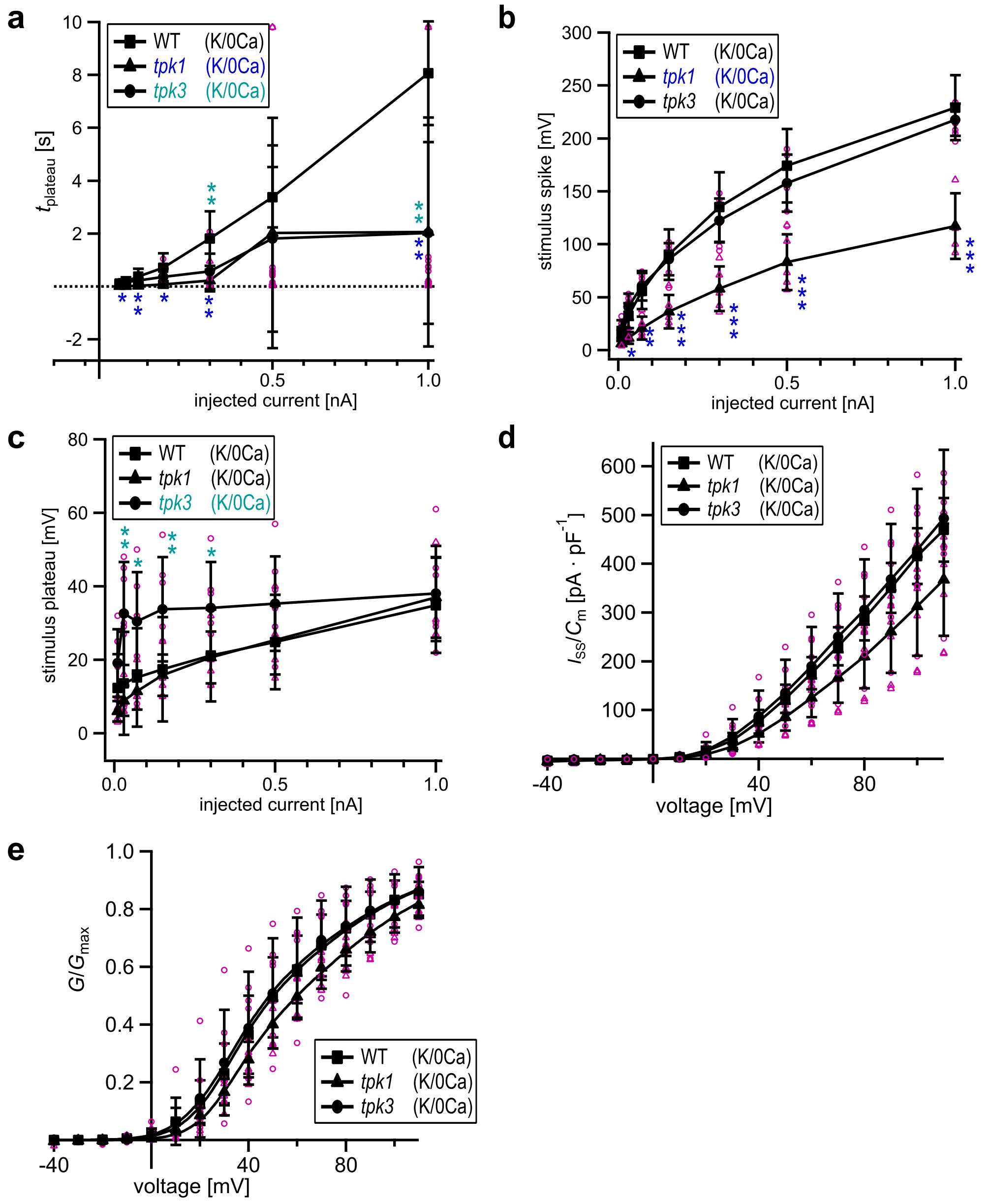

Supplementary Fig. 4: Effect of loss of TPK1 and TPK3 channel function on vacuolar electrical excitability. a-c, Voltage responses of vacuoles from wild type (WT, squares) and the single TPK loss-of-function mutants *tpk1* (triangles) and *tpk3* (circles) to current injections in the range of 10 up to 1000 pA. Amplitudes of triggered lifetime of the post-stimulus depolarized plateau phase (**a**), stimulus spike (**b**) and stimulus plateau (**c**) were plotted against the corresponding injected current. **d, e,** Steady-state current density (I_{ss}/C_m, **d**) and normalized conductance (G/G_{max}, **e**) determined for wild type (WT, squares), *tpk1* (triangles) and *tpk3* (circles) vacuoles were plotted against the corresponding voltage. Data points of all $G(V)$ curves (**b**) were described by Equation (1). In **a-c**, number of experiments was n = 7 for WT, n = 5 for

tpk1, n = 7 for *tpk3*. In **d, e,** number of experiments was n = 8 for WT, n = 7 for *tpk1* and *tpk3*. Closed symbols in **a-e** represent means ± standard deviation from experiments with individual vacuoles. Significant differences between wild type versus *tpk1* and wild type versus *tpk3* are displayed by blue and turquoise stars, respectively, according to the determined p-values (* = $p < 0.05$, ** = $p < 0.01$, *** = $p < 0.001$; one-way ANOVA followed by Bonferroni's post-hoc comparison test). Individual data points are given for *tpk1* and *tpk3* by open magenta triangles and circles, respectively. Wild type values (WT (K/0Ca)) in **a-c** are identical to those given in Fig. 1b, c, and wild type values (WT (K/0Ca)) in **d, e** are identical to those given in Supplementary Fig. 1c, d. Exact p-values determined for all data sets (**a-e)** are listed in Supplementary Data 1 and 2. Experiments in **a-e** were carried out under K^+-based standard solutions. Source data are provided as a Source Data file.

Supplementary Fig. 5

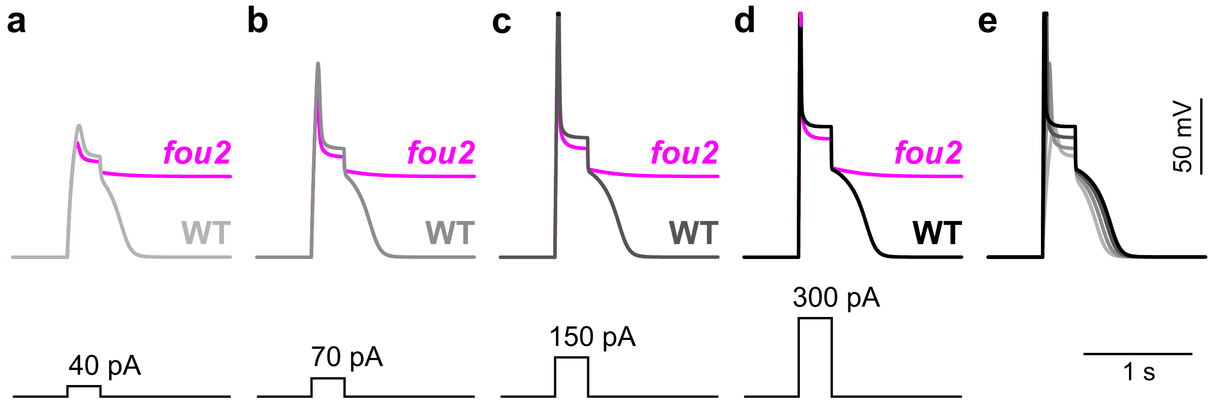

Supplementary Fig. 5: Simulated vacuolar electrical excitability without the contribution of TPKs. The electrical properties of the vacuole membrane were simulated with background and TPC1 conductance (black and grey curves, WT) or with background and *fou2* conductance (magenta curves), only. The application of four different current stimuli of 200 ms duration were tested: **a,** 40 pA; **b,** 70 pA; **c,** 150 pA; **d,** 300 pA. Panel **e** shows the superimposition of the curves obtained with the wild type TPC1 conductance (WT) in **a-d**. Source data are provided as a Source Data file.

Supplementary Fig. 6

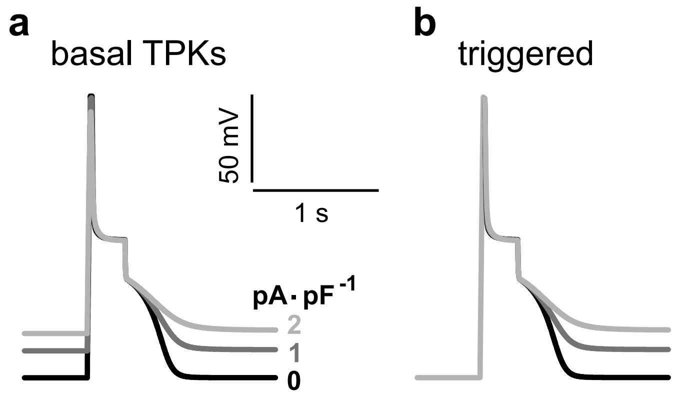

Supplementary Fig. 6: Simulated vacuolar electrical excitability with the contribution of TPKs. The electrical properties of the vacuole membrane were simulated with background, TPC1 and TPK conductance. **a,** TPKs have a basal background activity. **b,** The activity of the TPKs is triggered by the depolarization stimulus and then not further regulated. For each case, three different TPK current densities were tested: 0 pA·pF^{-1} (absence of TPKs; black curves), 1 pA·pF^{-1} (dark grey) and 2 pA·pF^{-1} (light grey). In each single case a 200 ms stimulus of 150 pA was applied to excite the membrane. Source data are provided as a Source Data file.

Supplementary Fig. 7

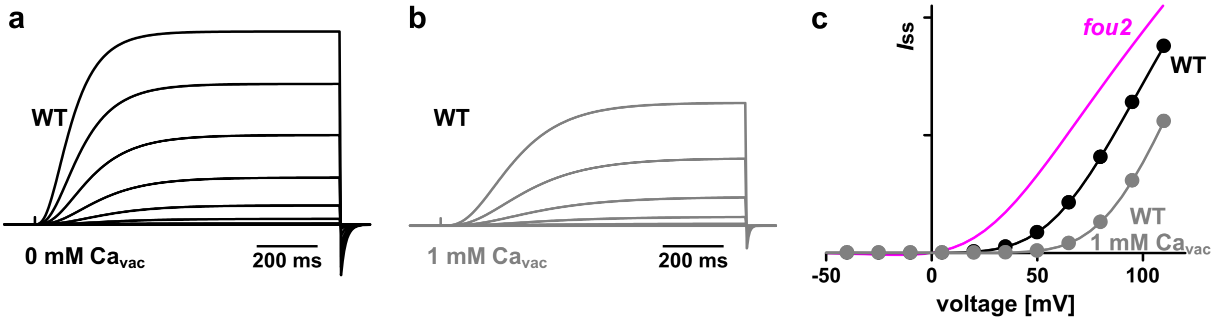

Supplementary Fig. 7: Simulation of voltage-clamp experiments with the *in silico* TPC1 channel. a, b, From a holding voltage of -40 mV, the membrane voltage was increased stepwise to voltages between +110 mV and -40 mV (15 mV steps, 1 s in duration). The displayed traces were simulated for wild type TPC1 channels (WT) with the presented gating scheme and **(a)** 0 mM and (**b**) 1 mM luminal Ca^{2+} (Ca_{vac}), respectively. **c,** Current-voltage characteristic of the steady-state currents (I_{ss}) with 0 mM (black) and 1 mM vacuolar Ca^{2+} (grey). The magenta curve illustrates the simulated TPC1-D454N (*fou2*) mutant with 0 mM vacuolar Ca^{2+}. Simulations were carried out under symmetrical solute conditions, i.e. equilibrium voltage of the permeating cation E_{cation} = 0 mV. Source data are provided as a Source Data file.

SUPPLEMENTARY NOTE 1

Computational simulation of electrical excitability at the tonoplast

The basis of a transient electric signal is the cable equation[1]. Compared to an axon or the plant phloem, the membrane surface of the vacuole of a plant cell is very small. In the time-scale of the observed action potential, the spatial equilibration across the tonoplast can therefore be considered as quasi instantaneous. In this case the cable equation simplifies to:

$$\frac{\partial}{\partial t}V(t) = \frac{1}{C_\mathrm{M}}\left[J_\mathrm{Stim}(t) - \sum_i g_i \cdot p_i\big(t, V(t)\big) \cdot [V(t) - E_i]\right] \qquad \text{Equation (S1)}$$

Here, the different parameters mean: (1) $V(t)$: tonoplast voltage at time t; unit: mV. (2) i: ion species to be considered. (3) E_i: equilibrium voltage for ion species i; unit: mV; (4) g_i: maximum conductance for ion species i; unit: pS·µm^{-2}; (5) $p_i(t, V(t))$: 'open channel probability' = activity of the conductance; $0 \leq p_i \leq 1$; (6) J_Stim: external stimulus to excite the system; unit: fA·µm^{-2}; (7) $C_\mathrm{M}=C_\mathrm{m}/A$: specific membrane capacity; unit: pF·µm^{-2}; C_m: membrane capacity of the vacuole; unit: pF. By the following re-definitions, the redundancy in system-specific parameters can be removed: (1) $\varepsilon_\mathrm{Stim}(t) = \frac{J_\mathrm{Stim}(t)}{C_\mathrm{M}}$ (unit: mV·s^{-1}) is a constant that describes the applied external stimulus. (2) $\varphi_i = \frac{g_i}{C_\mathrm{M}}$ (unit: s^{-1}) are channel-specific constants, which depend on the number of channels and the single channel conductance. With these definitions, the equation is:

$$\frac{\partial}{\partial t}V(t) = \varepsilon_\mathrm{Stim}(t) - \sum_i \varphi_i \cdot p_i\big(t, V(t)\big) \cdot [V(t) - E_i] \qquad \text{Equation (S2)}$$

To solve this equation numerically, the differential is approximated by a difference:

$$\frac{\partial}{\partial t}V(t) \rightarrow \frac{V(t+\Delta t) - V(t)}{\Delta t} \qquad \text{Equation (S3)}$$

Additionally, the t-Dimension is discretized in M points (index $m = 0\ldots M$) with the interval of Δt between two neighboring points: $V_m = V(t)$; $V_{m+1} = V(t + \Delta t)$; $p_{i,m} = p_i\big(t, V(t)\big)$. With

$$a_m = \Delta t \cdot \sum_i \varphi_i \cdot p_{i,m}, \qquad \text{Equation (S4)}$$

$$b_m = \Delta t \cdot \sum_i \varphi_i \cdot p_{i,m} \cdot E_i, \qquad \text{Equation (S5)}$$

$$\varepsilon_m = \Delta t \cdot \varepsilon_\mathrm{Stim}(t), \qquad \text{Equation (S6)}$$

the differential Equation (S2) converts into a linear equation that can be solved by iteration starting at $t = 0$ with $V_0 = V(0)$:

$$V_{m+1} = (1 - a_m) \cdot V_m + \varepsilon_m + b_m \qquad \text{Equation (S7)}$$

Mathematical description of channels and transporters in the vacuolar membrane

Background conductance

The background conductance, which is dominated by the activity of proton pumps, repolarizes the membrane voltage after electrical excitability. The background current (I_{BG}) was described by[2]:

$$I_{\text{BG}} = I_{\max} \cdot \frac{1 - exp\left\{-1.0 \cdot (V - V_0) \cdot \frac{F}{RT}\right\}}{1 + exp\left\{-1.0 \cdot (V - V_0) \cdot \frac{F}{RT}\right\}} \qquad \text{Equation (S8)}$$

The parameter V_0 denotes the voltage, at which the background current is zero. For the simulations in this study we chose $V_0 = -60\ mV$. I_{max} is the maximum current at positive voltages.

TPC1 conductance

The time- and voltage-dependent cation channel TPC1 confers excitability to the vacuolar membrane. Its delayed-activating behavior can be described mechanistically by four independent gates of two different types following the gating schemes $\mathrm{O}_1 \underset{d_1}{\overset{a_1}{\leftrightarrows}} \mathrm{C}_1$ and $\mathrm{O}_2 \underset{d_2}{\overset{a_2}{\leftrightarrows}} \mathrm{C}_2$.

With the following rate constants, the essential, experimentally-observed characteristics are satisfyingly reproduced (Supplementary Fig. 7):

$a_1 = s^{-1} \times \exp[0.45 \times V \times F/(RT) - 0.23 \times \ln(\{K + [Ca^{2+}]_{vac}\}/1\ mM)]$,

$d_1 = s^{-1} \times \exp[-0.81 \times V \times F/(RT) + 0.26 \times \ln(\{K + [Ca^{2+}]_{vac}\}/1\ mM) + 1.84]$,

$a_2 = s^{-1} \times \exp[0.5 \times V \times F/(RT) - 0.13 \times \ln(\{K + [Ca^{2+}]_{vac}\}/1\ mM) - 0.4]$,

$d_2 = s^{-1} \times \exp[-0.5 \times V \times F/(RT) + 0.12 \times \ln(\{K + [Ca^{2+}]_{vac}\}/1\ mM) + 3.0]$.

The parameter K was $K = 0.01$ mM for the wild type TPC1 channel; while the *fou2* (TPC1-D454N) mutant channel was represented by the value $K = 0.00001$ mM. Assuming that K^+ is the predominant permeating ion, the current through channels of the TPC1 type can be mathematically described by:

$$I_{\text{TPC}} = \sigma_{\text{TPC}} \cdot p_{\text{TPC}} \cdot (V - E_{\text{K}}) \qquad \text{Equation (S9)}$$

where σ_{TPC} is the maximal membrane conductance of the TPC1 channel, which depends on the single channel conductance and the number of channels, and p_{TPC} is the voltage-dependent open probability of the channels. σ_{TPC} was estimated from experimental data: In symmetric standard conditions ($E_K = 0$ mV), at a voltage of +100 mV, we measured a steady-state current density of $I_{ss}/C_m = 500\ pA \cdot pF^{-1}$, which corresponds to a current of $I_{TPC} = 10$ nA for a vacuole with membrane capacity of $C_m = 20$ pF. Thus, $\sigma_{TPC} = I_{TPC}/V = 10\ nA/100\ mV = 100\ nS$. With this value, the parameter φ_{TPC} can be determined, which is needed for equations S2, S4, and S5:

$\varphi_{\mathrm{TPC}} = g_{\mathrm{TPC}}/C_{\mathrm{M}} = \sigma_{\mathrm{TPC}}/A \times A/C_{\mathrm{m}} = 100$ nS/20 pF = 5000 s^{-1}, where A is the surface of the membrane.

TPK conductance as a security valve at very positive voltages

TPK channels were simulated as voltage-independent K^{+}-selective channels. The current through TPK channels can be expressed as

$$I_{\mathrm{TPK}} = \sigma_{\mathrm{TPK}} \cdot (V - E_{\mathrm{K}}) \qquad \text{Equation (S10)}$$

where σ_{TPK} is the conductance of this channel type in the membrane. As outlined in this study, a static σ_{TPK} cannot explain the observed phenomena (Supplementary Fig. 6a, b). Additionally, the depolarizing stimulus apparently did not activate the TPKs directly in the absence of TPC1-activity because neither *tpc1-2* vacuoles nor wild type vacuoles with blocked TPC1 showed the characteristic TPK-dependent post-stimulus plateau (Fig. 2a, b, red traces). Thus, instead, we needed to postulate that σ_{TPK} is stimulated in a TPC1-dependent manner. To simulate this effect, we chose the following possible scenario: σ_{TPK} is activated proportionally to the current flowing through TPC1 and decays in a voltage-dependent manner $d \sim \exp(-3 \times V \times F/(RT))$ with very low inactivation rates at positive voltages and increasing inactivation rates at more negative voltages. Because the parameter φ_{TPK} (used in equations S2, S4, and S5) is proportional to σ_{TPK}, $\varphi_{\mathrm{TPK}} = g_{\mathrm{TPK}}/C_{\mathrm{M}} = \sigma_{\mathrm{TPK}}/A \times A/C_{\mathrm{m}} = \sigma_{\mathrm{TPK}}/C_{\mathrm{m}}$, the transient activation can be directly modeled for φ_{TPK}.

Stimulus

The capacity of a typical vacuolar membrane was $C_{\mathrm{m}} = 20$ pF. The standard stimulus current in our experiments was $I_{\mathrm{Stim}} = 70$ pA. From these values, the parameter $\varepsilon_{\mathrm{Stim}}(t)$ for equations S2 and S6 can be deduced: $\varepsilon_{\mathrm{Stim}}(t) = J_{\mathrm{Stim}}(t)/C_{\mathrm{M}} = I_{\mathrm{Stim}}/A \times A/C_{\mathrm{m}} = I_{\mathrm{Stim}}/C_{\mathrm{m}} = 70$ pA/20 pF = 3.5 $\mathrm{V \cdot s^{-1}}$, where A is the membrane surface.

SUPPLEMENTARY REFERENCES

1. Hedrich, R., Salvador-Recatala, V. & Dreyer, I. Electrical wiring and long-distance plant communication. *Trends Plant Sci.* **21**, 376-387 (2016).

2. Schott, S. *et al.* Cooperation through competition-dynamics and microeconomics of a minimal nutrient trade system in arbuscular mycorrhizal Symbiosis. *Front Plant Sci.* **7**, 912 (2016).
