# Supplementary material for: Voltage-dependent gating of SV channel TPC1 confers vacuole excitability
Source: Nat Commun. 2019 Jun 14;10:2659. doi: 10.1038/s41467-019-10599-x (PMC6572840; doi:10.1038/s41467-019-10599-x)
Supplement: Supplementary file 3 — Description of Additional Supplementary Files [file 41467_2019_10599_MOESM3_ESM.docx]

**Description of Additional Supplementary Files**

File Name: Supplementary Data 1

Description: Statistical analysis of voltage clamp data.

File Name: Supplementary Data 2

Description: Statistical analysis of current clamp data.
